# Long-term trends in height, weight and body mass index of children and adolescents in Macao Special Administrative Region (China), 2005–2020

**DOI:** 10.1371/journal.pone.0351677

**Published:** 2026-06-12

**Authors:** Qingyuan Li, Yousong Yue

**Affiliations:** Sports Culture Research Center, Tianjin University of Sport, Tianjin, China; Sichuan University, CHINA

## Abstract

**Objective:**

To assess long-term trends in height, weight and body mass index (BMI) among children and adolescents from 2005 to 2020 in Macao Special Administrative Region (SAR), China.

**Methods:**

Height, weight and BMI data for Macao children and adolescents aged 6–18 years were obtained from the Physical Fitness Reports of Macao SAR Residents in 2005, 2010, 2015, and 2020. Sex-specific two-way analysis of variance was used to estimate the differences in means. The Bonferroni post hoc test was used for multiple comparisons.

**Results:**

During the entire period, the average height, weight and BMI increased by 2.1 cm (95% confidence interval (CI): 1.6 to 2.6 cm), 4.0 kg (95% CI: 3.2 to 4.8 kg), and 1.1 kg/m^2^ (95% CI: 0.8 to 1.3 kg/m^2^) for boys and 2.4 cm (95% CI: 1.9 to 2.9 cm), 2.6 kg (95% CI: 1.9 to 3.3 kg), and 0.5 kg/m^2^ (95% CI: 0.3 to 0.8 kg/m^2^) for girls, respectively (*p* < 0.001). Boys and girls in most age groups experienced significant increases. The greatest increases in height occurred between 2005 and 2010 in both sexes. The weight and BMI of boys have continued to increase. The weight and BMI of girls continued to increase until 2015, and thereafter declined.

**Conclusion:**

There were positive long-term trends in growth among Macao children and adolescents since 2005. Sex differences in changes of weight and BMI over the past five years may be related to the pandemic, and efforts are needed by governments and public health departments.

## Introduction

The long-term trends in growth is one of the most significant biological phenomena observed in humans over the past 200 years [1]. Height, weight, and body mass index (BMI) are important indicators of health and quality of living environment in children and adolescents, and they are highly predictive of lifelong health and well-being [2–3]. In children and adolescents, being shorter is associated with increased incidence and mortality of cardiovascular diseases, impaired cognitive development, and reduced educational performance in later life [4–5]; excessive weight and BMI gain increase the lifelong risks of being overweight, obesity, and suffering from various non-communicable diseases, such as cardiovascular diseases, coronary heart disease, heart failure, type II diabetes, and hypertension [3,6,7].

The long-term trend in growth is influenced by both genetic and environmental factors, with the most significant environmental factor being improvements in living conditions [1,8,9]. Since the 21st century, the positive trends in growth in many Western industrialized countries have slowed or stopped, suggesting that they may be nearing the full realization of their genetic potential, even as the economy continues to grow; however, positive trends in growth coexist with economic increase in some developing countries, particularly in emerging economies such as China, where significant increases in anthropometric indicators have been observed in many studies [2,9–13]. For developing countries, including China, analyzing long-term trends in growth provides important insights into monitoring dynamic changes in population health and public health conditions. Additionally, the biological evidence provided by these data can support governments in formulating efficient health policies and optimizing nutritional interventions for children and adolescents.

China was a country with diverse social, cultural, and geographical characteristics. Macao, located in southern China on the western side of the Pearl River Estuary, was one of the Chinese Special Administrative Regions (SARs). Macao had been a Chinese territory since ancient times, but after Portugal established its first official settlement there in the 1660s, Macao gradually fell under Portuguese colonial control, and sovereignty of China over the region became nominal. It was not until December 20, 1999, that the Chinese government resumed the exercise of sovereignty over Macao, and the Macao SAR was established [14]. Although China implemented a socialist system, Macao continued to implement a capitalist system and enjoyed a high degree of autonomy in all matters except foreign and military affairs, which was the principle of “one country, two systems.” After more than four centuries of cultural integration between East and West, Macao has become a unique place and an interesting location for studying long-term trends. Given Macao’s unique political system and diverse population, large-scale anthropometric surveys conducted nationwide in China generally did not sample Macao [11,12], resulting in a lack of anthropometric evidence for children and adolescents in Macao. Choi et al. [15] reported significant increases in height and weight among preschool children aged 3–6 years in Macao from 2002 to 2020, but no significant changes in BMI. A study reported data on the height and weight of children and adolescents aged 6–18 years in Macao from 2005 to 2020 [16]. However, these data were crude and lacked sufficient detail. Currently, no studies have comprehensively estimated the magnitude and pace of trends in growth among children and adolescents in Macao, which reflect changes in Macao’s socioeconomic conditions and public health services.

The Macao Citizens Physical Fitness Monitoring and Assessment was organized by the Sports Bureau of Macao SAR and supported by the General Administration of Sport of China. Since 2005, it has been conducted every five years and is the largest and most representative anthropometric survey of Macao population. Therefore, by the four Physical Fitness Reports of Macao SAR Residents from 2005 to 2020 [17–20], this paper aimed to examine the direction and pace of trends in height, weight and BMI among Macao children and adolescents aged 6–18 years.

## Methods

### Study design and subjects

Since 2005, the Sports Bureau, the Education and Youth Development Bureau, the Health Bureau and the Social Welfare Bureau of Macao SAR, the Macao Polytechnic University, and the General Administration of Sport of China have been conducting the Macao Citizens Physical Fitness Monitoring and Assessment. The sampling and measurement procedures are described in detail elsewhere [17–20]. A stratified random cluster sampling method was used to divide schools into three areas based on the location of their main campuses: the northern area, the central area, and the southern area. Then, 2–3 schools were randomly selected from each area, and sampling was conducted at the class level. The sample was divided into male and female groups based on sex, with one year forming one age group, for a total of 26 age groups. 55 students were selected from each sex and age group in each area. Participants must be in good health and can perform simple physical activities. A total of 17,984, 17,982 and 17,979 records for height, weight and BMI among children and adolescents aged 6–18 years were obtained, respectively. The sample sizes by test, sex, and wave are shown in Table 1. The sample sizes by test, wave, sex and age are shown in Si Table.The sample size for each age group ranged from 100 to 200. Written informed consent was obtained from all participants’ parents. This study was exempt from ethical approval due to the use of publicly available, anonymized summary data.

**Table 1:** Sample sizes among Macao children and adolescents by wave and sex.

Test	Boys				Girls				Total			
	2005	2010	2015	2020	2005	2010	2015	2020	2005	2010	2015	2020
Height (cm)	2374	2223	2495	2582	2144	2107	1949	2110	4518	4330	4444	4692
Weight (kg)	2374	2223	2494	2584	2144	2105	1948	2110	4518	4328	4442	4694
Body mass index (kg/m^2^)	2374	2223	2493	2582	2144	2105	1948	2110	4518	4328	4441	4692

### Measurements

The testing was conducted from January to April of 2005–2015 and from September to November of 2020. The sampled schools were nearly identical across all waves. The sampling methods and measurement procedures were consistent across all testing sites, and the same brand of testing equipment (Jianmin II, Beijing) was used. Instruments were calibrated before each measurement. All staff received standardized training and passed the assessment. Height was measured by a stadiometer, accurate to 0.1 cm. Participants stand barefoot with their backs toward the pillar on the base plate of the stadiometer. Their trunks were naturally straight, their heads were upright, and their eyes looked straight ahead. Weight was measured by a electronic scale, accurate to 0.1 kg. Participants wore shorts and bare feet, stood naturally in the center of the platform of the electronic scale, and kept their bodies steady. BMI was calculated as body mass in kilograms divided by body height in meters squared.

### Statistical analysis

Height, weight and BMI are expressed as the means and standard deviations. Trends were estimated separately for boys and girls. Two-way analysis of variance (ANOVA) with age and wave as main factors, and age-by-wave as the interaction, was used to estimate the differences in means. The partial eta squared (η^2^) was used to measure the standardized effect sizes for each factor. The thresholds for small, medium, and large ESs were 0.01, 0.06, and 0.14 or greater, respectively [21]. The Bonferroni post hoc test was used for multiple comparisons. The differences between adjacent waves were expressed as the mean and 95% confidence interval (CI). The level of statistical significance was set at 0.05. All analyses were conducted using GraphPad Prism 9.3.1 (GraphPad Software, Inc., CA, USA).

## Results

### Trends in height

**Fig 1:**
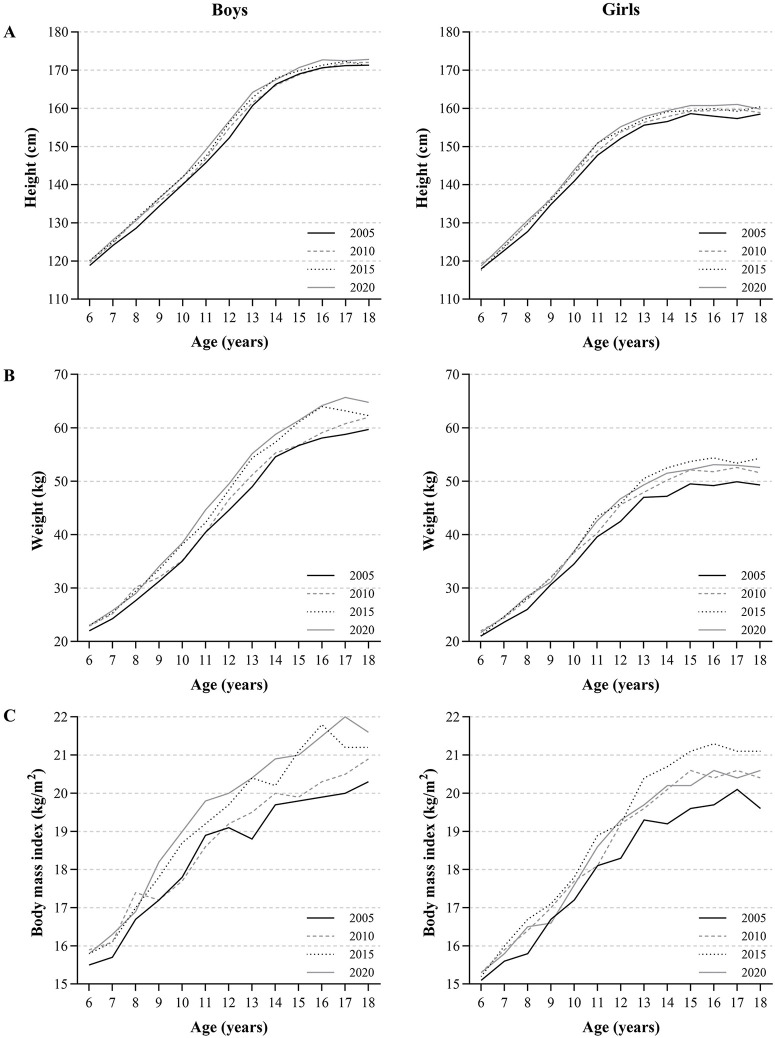
Means in height, weight and body mass index among Macao children and adolescents by wave, sex, and age. **(A)**: height; **(B)**: weight; **(C)**: body mass index.

The means in height among Macao children and adolescents by wave, sex and age are shown in Fig 1A. The results of the two-way ANOVA analysis for height showed that there were large effects for age (boys: *F* = 6460.729, *p* < 0.001, partial η^2^ = 0.8698; girls: *F* = 3983.205, *p* < 0.001, partial η^2^ = 0.8356), small effects for waves (boys: *F* = 49.876, *p* < 0.001, partial η^2^ = 0.0017; girls: *F* = 61.692, *p* < 0.001, partial η^2^ = 0.0032) and the age-by-wave interaction (boys: *F* = 1.778, *p* = 0.003, partial η^2^ = 0.0007; girls: *F* = 1.438, *p* = 0.044, partial η^2^ = 0.0009). During the entire period, the average height among boys and girls increased by 2.1 cm (95% CI: 1.6 to 2.6 cm) and 2.4 cm (95% CI: 1.9 to 2.9 cm), respectively (*p* < 0.001). The height of boys aged 8–13, 16 years and girls aged 7–8, 10–17 years increased significantly (ranging from 1.8 to 4.4 cm). In terms of periods, the average height of boys increased by 0.8 cm, 0.8 cm, and 0.5 cm during the 2005–2010, 2010–2015, and 2015–2020 periods, respectively. For girls, the height increments were 1.4 cm, 0.3 cm, and 0.7 cm, respectively, in three periods. The height growth trends for both boys and girls have slowed. Most significant increases occurred in some age groups from 2005 to 2010. Trends in height declined for both sexes (Table 2).

**Table 2:** Means, SDs, and differences between waves of height among Macao children and adolescent.

Sex	Age (years)	Surveys				Differences and 95% CI in means			
		2005	2010	2015	2020	2010−2005	2015−2010	2020−2015	2020−2005
		M ± SD	M ± SD	M ± SD	M ± SD				
Boys	6	118.8 ± 4.5	119.6 ± 5.0	120.1 ± 5.1	120.0 ± 5.5	0.8 (−1.3, 2.9)	0.5 (−1.6, 2.6)	−0.1 (−1.8, 1.6)	1.2 (−0.5, 2.9)
	7	124.1 ± 5.4	124.7 ± 5.6	125.0 ± 5.1	125.5 ± 6.2	0.6 (−1.1, 2.3)	0.3 (−1.3, 1.9)	0.5 (−1.1, 2.1)	1.4 (−0.2, 3.0)
	8	128.6 ± 5.5	130.9 ± 6.3	131.1 ± 5.8	130.6 ± 6.0	2.3 (0.5, 4.1)**	0.2 (−1.6, 2.0)	−0.5 (−2.1, 1.1)	2.0 (0.4, 3.6)**
	9	134.4 ± 5.4	135.7 ± 6.2	136.6 ± 6.5	136.5 ± 6.1	1.3 (−0.4, 3.0)	0.9 (−0.8, 2.6)	−0.1 (−1.8, 1.6)	2.1 (0.3, 3.9)**
	10	140.1 ± 6.6	140.2 ± 6.2	142.1 ± 6.5	142.0 ± 7.0	0.1 (−1.7, 1.9)	1.9 (0.0, 3.8)*	−0.1 (−1.9, 1.7)	1.9 (0.1, 3.7)*
	11	145.8 ± 7.7	146.8 ± 7.5	147.4 ± 7.7	149.2 ± 8.2	1.0 (−0.9, 2.9)	0.6 (−1.3, 2.5)	1.8 (0.0, 3.6)	3.4 (1.6, 5.2)***
	12	152.3 ± 8.1	154.9 ± 8.4	156.3 ± 7.8	156.7 ± 8.7	2.6 (0.8, 4.4)***	1.4 (−0.3, 3.1)	0.4 (−1.3, 2.1)	4.4 (2.7, 6.1)***
	13	160.7 ± 7.8	161.5 ± 7.5	162.8 ± 7.8	164.2 ± 7.7	0.8 (−1.0, 2.6)	1.3 (−0.5, 3.1)	1.4 (−0.4, 3.2)	3.5 (1.7, 5.3)***
	14	166.3 ± 6.7	166.0 ± 6.1	167.9 ± 6.5	167.5 ± 7.0	−0.3 (−2.2, 1.6)	1.9 (0.1, 3.7)*	−0.4 (−2.1, 1.3)	1.2 (−0.6, 3.0)
	15	169.0 ± 6.0	168.8 ± 6.7	169.9 ± 6.1	170.7 ± 5.8	−0.2 (−2.0, 1.6)	1.1 (−0.7, 2.9)	0.8 (−1.0, 2.6)	1.7 (−0.1, 3.5)
	16	170.6 ± 5.9	170.5 ± 6.3	171.3 ± 5.8	172.7 ± 6.0	−0.1 (−2.0, 1.8)	0.8 (−1.0, 2.6)	1.4 (−0.4, 3.2)	2.1 (0.3, 3.9)*
	17	171.2 ± 6.3	171.9 ± 5.5	172.3 ± 6.0	172.5 ± 6.0	0.7 (−1.1, 2.5)	0.4 (−1.3, 2.1)	0.2 (−1.5, 1.9)	1.3 (−0.5, 3.1)
	18	171.3 ± 5.4	172.0 ± 5.9	171.1 ± 6.2	172.8 ± 6.0	0.7 (−1.3, 2.7)	−0.9 (−2.9, 1.1)	1.7 (−0.2, 3.6)	1.5 (−0.4, 3.4)
	Total	150.2 ± 19.7	151.0 ± 19.0	151.8 ± 19.7	152.4 ± 20.3	0.8 (0.3, 1.3)***	0.8 (0.3, 1.3)***	0.5 (0.1, 1.0)*	2.1 (1.6, 2.6)***
Girls	6	117.9 ± 5.0	119.3 ± 4.7	117.6 ± 5.1	118.7 ± 5.6	1.4 (−0.6, 3.4)	−1.7 (−3.8, 0.4)	1.1 (−0.7, 2.9)	0.8 (−0.9, 2.5)
	7	122.8 ± 5.0	123.5 ± 6.1	123.9 ± 5.4	124.6 ± 5.3	0.7 (−1.0, 2.4)	0.4 (−1.3, 2.1)	0.7 (−0.9, 2.3)	1.8 (0.1, 3.5)*
	8	127.7 ± 6.3	129.9 ± 6.4	129.8 ± 6.7	130.6 ± 6.8	2.2 (0.4, 4.0)**	−0.1 (−2.0, 1.8)	0.8 (−1.0, 2.6)	2.9 (1.1, 4.7)***
	9	134.8 ± 6.3	136.6 ± 6.0	135.9 ± 6.8	136.2 ± 6.7	1.8 (0.1, 3.5)*	−0.7 (−2.5, 1.1)	0.3 (−1.5, 2.1)	1.4 (−0.3, 3.1)
	10	140.9 ± 6.6	142.9 ± 7.2	143.1 ± 7.2	143.9 ± 7.4	2.0 (0.2, 3.8)*	0.2 (−1.6, 2.0)	0.8 (−1.0, 2.6)	3.0 (1.2, 4.8)***
	11	147.7 ± 6.0	148.8 ± 7.4	150.9 ± 6.6	150.9 ± 6.7	1.1 (−0.7, 2.9)	2.1 (0.3, 3.9)*	0.0 (−1.8, 1.8)	3.2 (1.4, 5.0)***
	12	152.1 ± 6.0	153.8 ± 6.2	154.1 ± 7.1	155.2 ± 5.7	1.7 (0.0, 3.4)*	0.3 (−1.5, 2.1)	1.1 (−0.6, 2.8)	3.1 (1.5, 4.7)***
	13	155.6 ± 6.0	156.3 ± 5.6	157.1 ± 5.3	157.8 ± 5.7	0.7 (−1.0, 2.4)	0.8 (−1.1, 2.7)	0.7 (−1.2, 2.6)	2.2 (0.5, 3.9)**
	14	156.5 ± 5.8	157.8 ± 5.1	159.1 ± 5.2	159.4 ± 5.7	1.3 (−0.4, 3.0)	1.3 (−0.4, 3.0)	0.3 (−1.5, 2.1)	2.9 (1.1, 4.7)***
	15	158.6 ± 5.4	159.2 ± 5.2	159.4 ± 5.7	160.7 ± 4.8	0.6 (−1.0, 2.2)	0.2 (−1.5, 1.9)	1.3 (−0.5, 3.1)	2.1 (0.4, 3.8)**
	16	157.9 ± 5.9	159.4 ± 6.0	159.9 ± 5.9	160.7 ± 5.4	1.5 (−0.1, 3.1)	0.5 (−1.2, 2.2)	0.8 (−1.0, 2.6)	2.8 (1.1, 4.5)***
	17	157.3 ± 5.2	159.7 ± 4.9	159.2 ± 5.5	161.0 ± 5.4	2.4 (0.8, 4.0)***	−0.5 (−2.1, 1.1)	1.8 (0.2, 3.4)*	3.7 (2.1, 5.3)***
	18	158.5 ± 5.7	158.9 ± 5.6	160.4 ± 5.1	159.8 ± 5.1	0.4 (−1.3, 2.1)	1.5 (−0.1, 3.1)	−0.6 (−2.3, 1.1)	1.3 (−0.5, 3.1)
	Total	145.3 ± 15.3	146.6 ± 14.6	147.0 ± 16.0	147.7 ± 16.0	1.4 (0.9, 1.9)***	0.3 (−0.2, 0.8)	0.7 (0.2, 1.2)*	2.4 (1.9, 2.9)***

Two-way analysis of variance with age and wave as main factors, and age-by-wave as the interaction. Bonferroni post hoc test was used for multiple comparisons. * represents *p* < 0.05, ** represents *p* < 0.01, and *** represents *p* < 0.001. M, mean; SD, standard deviation; CI, confidence interval.

### Trends in weight

The means in weight among Macao children and adolescents by wave, sex and age are shown in Fig 1B. The results of the two-way ANOVA analysis for weight showed that there were large effects for age (boys: *F* = 1462.299, *p* < 0.001, partial η^2^ = 0.6293; girls: *F* = 1237.470, *p* < 0.001, partial η^2^ = 0.6309), small effects for waves (boys: *F* = 73.199, *p* < 0.001, partial η^2^ = 0.0079; girls: *F* = 57.820, *p* < 0.001, partial η^2^ = 0.0074) and the age-by-wave interaction (boys: *F* = 2.117, *p* < 0.001, partial η^2^ = 0.0027; girls: *F* = 1.602, *p* = 0.013, partial η^2^ = 0.0025). During the entire period, the average weight among boys and girls increased by 4.0 kg (95% CI: 3.2 to 4.8 kg) and 2.6 kg (95% CI: 1.9 to 3.3 kg), respectively (*p* < 0.001). The weight of boys aged 9−18 years and girls aged 8, 11−12, 14−18 years increased significantly (ranging from 2.5 to 6.9 kg). In terms of periods, the average weight of boys increased by 1.2 kg, 1.9 kg, and 0.9 kg during the 2005−2010, 2010−2015, and 2015−2020 periods, respectively. For girls, the weight increments were 1.9 kg, 1.2 kg, and −0.5 kg, respectively, in three periods. Trends in weight declined for girls, but remained stable for boys (Table 3).

**Table 3:** Means, SDs, and differences between waves of weight among Macao children and adolescent.

Sex	Age (years)	Surveys				Differences and 95% CI in means			
		2005	2010	2015	2020	2010−2005	2015−2010	2020−2015	2020−2005
		M ± SD	M ± SD	M ± SD	M ± SD				
Boys	6	22.0 ± 3.6	22.9 ± 4.8	23.0 ± 4.9	23.0 ± 5.1	0.9 (−2.5, 4.3)	0.1 (−3.3, 3.5)	0.0 (−2.7, 2.7)	1.0 (−1.8, 3.8)
	7	24.3 ± 5.0	25.2 ± 5.4	25.4 ± 5.4	25.8 ± 5.4	0.9 (−1.8, 3.6)	0.2 (−2.4, 2.8)	0.4 (−2.1, 2.9)	1.5 (−1.1, 4.1)
	8	27.7 ± 6.2	30.2 ± 8.4	29.5 ± 7.5	29.0 ± 6.7	2.5 (−0.4, 5.4)	−0.7 (−3.6, 2.2)	−0.5 (−3.1, 2.1)	1.3 (−1.3, 3.9)
	9	31.3 ± 7.2	32.0 ± 8.4	33.5 ± 8.1	34.1 ± 8.3	0.7 (−2.1, 3.5)	1.5 (−1.2, 4.2)	0.6 (−2.1, 3.3)	2.8 (0.0, 5.6)*
	10	35.1 ± 8.1	35.2 ± 8.8	38.2 ± 9.6	38.5 ± 10.2	0.1 (−2.8, 3.0)	3.0 (0.0, 6.0)*	0.3 (−2.6, 3.2)	3.4 (0.6, 6.2)**
	11	40.5 ± 10.7	40.5 ± 10.4	42.3 ± 11.6	44.7 ± 13.7	0.0 (−3.1, 3.1)	1.8 (−1.3, 4.9)	2.4 (−0.6, 5.4)	4.2 (1.3, 7.1)***
	12	44.6 ± 11.6	46.6 ± 12.9	48.4 ± 11.5	49.5 ± 13.8	2.0 (−0.8, 4.8)	1.8 (−1.0, 4.6)	1.1 (−1.7, 3.9)	4.9 (2.1, 7.7)***
	13	49.0 ± 11.5	51.2 ± 11.7	54.4 ± 13.1	55.2 ± 13.9	2.2 (−0.7, 5.1)	3.2 (0.3, 6.1)*	0.8 (−2.0, 3.6)	6.2 (3.4, 9.0)***
	14	54.6 ± 11.9	55.3 ± 11.9	57.3 ± 13.2	58.8 ± 13.6	0.7 (−2.3, 3.7)	2.0 (−0.9, 4.9)	1.5 (−1.3, 4.3)	4.2 (1.3, 7.1)***
	15	56.7 ± 12.6	56.8 ± 11.0	61.1 ± 14.3	61.4 ± 12.6	0.1 (−2.8, 3.0)	4.3 (1.4, 7.2)***	0.3 (−2.6, 3.2)	4.7 (1.7, 7.7)***
	16	58.1 ± 10.0	59.1 ± 11.7	64.0 ± 13.2	64.2 ± 14.8	1.0 (−2.0, 4.0)	4.9 (2.0, 7.8)***	0.2 (−2.7, 3.1)	6.1 (3.1, 9.1)***
	17	58.8 ± 10.8	60.8 ± 9.9	63.2 ± 12.5	65.7 ± 14.2	2.0 (−0.9, 4.9)	2.4 (−0.4, 5.2)	2.5 (−0.3, 5.3)	6.9 (3.9, 9.9)***
	18	59.7 ± 9.5	62.0 ± 11.1	62.3 ± 10.9	64.8 ± 12.0	2.3 (−0.9, 5.5)	0.3 (−2.9, 3.5)	2.5 (−0.6, 5.6)	5.1 (2.0, 8.2)***
	Total	43.3 ± 16.4	44.5 ± 16.7	46.4 ± 18.3	47.3 ± 19.1	1.2 (0.4, 2.0)***	1.9 (1.1, 2.7)***	0.9 (0.2, 1.7)**	4.0 (3.2, 4.8)***
Girls	6	21.0 ± 3.2	22.0 ± 3.8	21.1 ± 3.6	21.7 ± 4.3	1.0 (−1.8, 3.8)	−0.9 (−3.8, 2.0)	0.6 (−1.9, 3.1)	0.7 (−1.7, 3.1)
	7	23.6 ± 4.7	24.4 ± 4.8	24.7 ± 5.3	24.6 ± 5.2	0.8 (−1.6, 3.2)	0.3 (−2.0, 2.6)	−0.1 (−2.4, 2.2)	1.0 (−1.4, 3.4)
	8	26.0 ± 5.3	27.9 ± 7.0	28.2 ± 6.3	28.5 ± 6.5	1.9 (−0.6, 4.4)	0.3 (−2.3, 2.9)	0.3 (−2.3, 2.9)	2.5 (0.0, 5.0)*
	9	30.6 ± 7.0	32.0 ± 7.1	31.9 ± 7.4	31.1 ± 7.0	1.4 (−1.0, 3.8)	−0.1 (−2.6, 2.4)	−0.8 (−3.3, 1.7)	0.5 (−1.9, 2.9)
	10	34.5 ± 8.8	36.6 ± 8.9	36.7 ± 9.3	36.9 ± 9.9	2.1 (−0.4, 4.6)	0.1 (−2.4, 2.6)	0.2 (−2.3, 2.7)	2.4 (−0.1, 4.8)
	11	39.6 ± 8.1	40.3 ± 8.9	43.4 ± 9.6	42.6 ± 9.7	0.7 (−1.8, 3.2)	3.1 (0.6, 5.6)**	−0.8 (−3.3, 1.7)	3.0 (0.5, 5.5)***
	12	42.5 ± 8.1	45.6 ± 9.6	45.8 ± 9.9	46.7 ± 9.1	3.1 (0.8, 5.4)**	0.2 (−2.2, 2.6)	0.9 (−1.5, 3.3)	4.2 (1.9, 6.5)***
	13	47.0 ± 10.2	47.9 ± 9.4	50.5 ± 10.4	49.3 ± 9.4	0.9 (−1.5, 3.3)	2.6 (0.0, 5.1)*	−1.2 (−3.8, 1.4)	2.3 (−0.1, 4.7)
	14	47.2 ± 8.6	50.2 ± 8.8	52.5 ± 9.4	51.5 ± 9.8	3.0 (0.6, 5.4)**	2.3 (−0.1, 4.7)	−1.0 (−3.5, 1.5)	4.3 (1.8, 6.8)***
	15	49.5 ± 7.7	52.1 ± 9.5	53.7 ± 9.3	52.2 ± 8.8	2.6 (0.3, 4.9)*	1.6 (−0.8, 4.0)	−1.5 (−4.0, 1.0)	2.7 (0.4, 5.0)*
	16	49.2 ± 8.0	51.8 ± 8.2	54.4 ± 9.1	53.1 ± 9.6	2.6 (0.4, 4.8)*	2.6 (0.2, 5.0)*	−1.3 (−3.8, 1.2)	3.9 (1.6, 6.2)***
	17	49.9 ± 8.0	52.6 ± 9.4	53.4 ± 10.0	53.0 ± 9.6	2.7 (0.4, 5.0)**	0.8 (−1.5, 3.1)	−0.4 (−2.7, 1.9)	3.1 (0.8, 5.4)**
	18	49.3 ± 6.8	51.6 ± 8.7	54.3 ± 8.4	52.6 ± 8.7	2.3 (0.0, 4.6)	2.7 (0.4, 5.0)*	−1.7 (−4.1, 0.7)	3.3 (0.9, 5.7)**
	Total	39.2 ± 12.8	41.2 ± 13.4	42.4 ± 14.7	41.8 ± 14.2	1.9 (1.3, 2.6)***	1.2 (0.5, 1.9)***	−0.5 (−1.2, 0.2)	2.6 (1.9, 3.3)***

Two-way analysis of variance with age and wave as main factors, and age-by-wave as the interaction. Bonferroni post hoc test was used for multiple comparisons. * represents *p* < 0.05, ** represents *p* < 0.01, and *** represents *p* < 0.001. M, mean; SD, standard deviation; CI, confidence interval.

### Trends in BMI

The means in BMI among Macao children and adolescents by wave, sex and age are shown in Fig 1C. The results of the two-way ANOVA analysis for BMI showed that there were large effects for age (boys: *F* = 199.491, *p* < 0.001, partial η^2^ = 0.1947; girls: *F* = 256.521, *p* < 0.001, partial η^2^ = 0.2677), small to medium effects for waves (boys: *F* = 45.890, *p* < 0.001, partial η^2^ = 0.0112; girls: *F* = 33.263, *p* < 0.001, partial η^2^ = 0.0088) and no to small effects for the age-by-wave interaction (boys: *F* = 1.699, *p* = 0.006, partial η^2^ = 0.0050; girls: *F* = 1.055, *p* = 0.380, partial η^2^ = 0.0033). During the entire period, the average BMI among boys and girls increased by 1.1 kg/m^2^ (95% CI: 0.8 to 1.3 kg/m^2^) and 0.5 kg/m^2^ (95% CI: 0.3 to 0.8 kg/m^2^), respectively (*p* < 0.001). The BMI of boys aged 9−18 years and girls aged 12, 14, 16, 18 years increased significantly (ranging from 0.9 to 2.0 kg/m^2^). In terms of periods, the average weight of boys increased by 0.3 kg/m^2^, 0.5 kg/m^2^, and 0.3 kg/m^2^ during the 2005−2010, 2010−2015, and 2015−2020 periods, respectively. For girls, the weight increments were 0.5 kg/m^2^, 0.4 kg/m^2^, and −0.4 kg/m^2^, respectively, in three periods. Trends in BMI declined even reversed for girls, but remained stable for boys (Table 4).

**Table 4:** Means, SDs, and differences between waves of body mass index among Macao children and adolescent.

Sex	Age (years)	Surveys				Differences and 95% CI in means			
		2005	2010	2015	2020	2010−2005	2015−2010	2020−2015	2020−2005
		M ± SD	M ± SD	M ± SD	M ± SD				
Boys	6	15.5 ± 1.8	15.9 ± 2.5	15.8 ± 2.3	15.8 ± 2.4	0.4 (−0.8, 1.6)	−0.1 (−1.3, 1.1)	0.0 (−0.9, 0.9)	0.3 (−0.6, 1.2)
	7	15.7 ± 2.4	16.1 ± 3.0	16.1 ± 2.6	16.3 ± 2.6	0.4 (−0.5, 1.3)	0.0 (−0.9, 0.9)	0.2 (−0.7, 1.1)	0.6 (−0.3, 1.5)
	8	16.7 ± 2.9	17.4 ± 3.7	17.0 ± 3.2	16.9 ± 2.9	0.7 (−0.3, 1.7)	−0.4 (−1.4, 0.6)	−0.1 (−1.0, 0.8)	0.2 (−0.7, 1.1)
	9	17.2 ± 3.1	17.2 ± 3.4	17.8 ± 3.2	18.2 ± 3.5	0.0 (−0.9, 0.9)	0.6 (−0.3, 1.5)	0.4 (−0.5, 1.3)	1.0 (0.0, 2.0)*
	10	17.8 ± 3.3	17.7 ± 3.4	18.7 ± 3.6	19.0 ± 4.7	−0.1 (−1.1, 0.9)	1.0 (0.0, 2.0)	0.3 (−0.7, 1.3)	1.2 (0.2, 2.2)**
	11	18.9 ± 3.7	18.6 ± 3.7	19.2 ± 3.9	19.8 ± 4.5	−0.3 (−1.3, 0.7)	0.6 (−0.5, 1.7)	0.6 (−0.4, 1.6)	0.9 (0.1, 1.7)*
	12	19.1 ± 3.9	19.2 ± 3.9	19.7 ± 3.9	20.0 ± 4.3	0.1 (−0.9, 1.1)	0.5 (−0.5, 1.5)	0.3 (−0.6, 1.2)	0.9 (0.0, 1.8)*
	13	18.8 ± 3.5	19.5 ± 3.6	20.4 ± 4.1	20.4 ± 4.3	0.7 (−0.3, 1.7)	0.9 (−0.1, 1.8)	0.0 (−1.0, 1.0)	1.6 (0.6, 2.6)***
	14	19.7 ± 3.6	20.0 ± 4.0	20.2 ± 3.9	20.9 ± 4.3	0.3 (−0.7, 1.3)	0.2 (−0.8, 1.2)	0.7 (−0.3, 1.7)	1.2 (0.2, 2.2)**
	15	19.8 ± 4.5	19.9 ± 3.1	21.1 ± 4.5	21.0 ± 3.9	0.1 (−0.9, 1.1)	1.2 (0.2, 2.2)**	−0.1 (−1.1, 0.9)	1.2 (0.2, 2.2)*
	16	19.9 ± 2.9	20.3 ± 3.6	21.8 ± 4.2	21.5 ± 4.6	0.4 (−0.6, 1.4)	1.5 (0.5, 2.5)***	−0.3 (−1.3, 0.7)	1.6 (0.6, 2.6)***
	17	20.0 ± 3.3	20.5 ± 3.1	21.2 ± 3.9	22.0 ± 4.4	0.5 (−0.5, 1.5)	0.7 (−0.2, 1.6)	0.8 (−0.1, 1.7)	2.0 (1.0, 3.0)***
	18	20.3 ± 3.1	20.9 ± 3.4	21.2 ± 3.4	21.6 ± 3.7	0.6 (−0.5, 1.7)	0.3 (−0.8, 1.4)	0.4 (−0.6, 1.4)	1.3 (0.3, 2.3)**
	Total	18.4 ± 3.7	18.7 ± 3.8	19.2 ± 4.1	19.5 ± 4.4	0.3 (0.0, 0.6)*	0.5 (0.3, 0.8)***	0.3 (0.0, 0.6)*	1.1 (0.8, 1.3)***
Girls	6	15.1 ± 1.5	15.3 ± 1.9	15.2 ± 1.8	15.3 ± 2.1	0.2 (−0.8, 1.2)	−0.1 (−1.2, 1.0)	0.1 (−0.8, 1.0)	0.2 (−0.7, 1.1)
	7	15.6 ± 2.4	15.9 ± 2.2	16.0 ± 2.5	15.8 ± 2.5	0.3 (−0.6, 1.2)	0.1 (−0.8, 1.0)	−0.2 (−1.1, 0.7)	0.2 (−0.7, 1.1)
	8	15.8 ± 2.3	16.4 ± 2.9	16.7 ± 3.0	16.5 ± 2.5	0.6 (−0.3, 1.5)	0.3 (−0.7, 1.3)	−0.2 (−1.1, 0.8)	0.7 (−0.2, 1.6)
	9	16.7 ± 2.9	17.0 ± 3.0	17.1 ± 2.8	16.6 ± 2.7	0.3 (−0.6, 1.2)	0.1 (−0.8, 1.0)	−0.5 (−1.4, 0.4)	−0.1 (−1.0, 0.8)
	10	17.2 ± 3.4	17.7 ± 3.3	17.8 ± 3.3	17.6 ± 3.4	0.5 (−0.4, 1.4)	0.1 (−0.8, 1.0)	−0.2 (−1.1, 0.7)	0.4 (−0.5, 1.3)
	11	18.1 ± 3.1	18.1 ± 3.1	18.9 ± 3.4	18.6 ± 3.5	0.0 (−0.9, 0.9)	0.8 (−0.1, 1.7)	−0.3 (−1.2, 0.7)	0.5 (−0.4, 1.4)
	12	18.3 ± 3.1	19.2 ± 3.3	19.2 ± 3.3	19.3 ± 3.3	0.9 (0.0, 1.8)*	0.0 (−0.9, 0.9)	0.1 (−0.8, 1.0)	1.0 (0.1, 1.9)*
	13	19.3 ± 3.6	19.6 ± 3.3	20.4 ± 3.6	19.7 ± 3.2	0.3 (−0.6, 1.2)	0.8 (−0.2, 1.8)	−0.7 (−1.7, 0.3)	0.4 (−0.5, 1.3)
	14	19.2 ± 3.0	20.1 ± 3.2	20.7 ± 3.4	20.2 ± 3.6	0.9 (0.0, 1.7)*	0.6 (−0.3, 1.5)	−0.5 (−1.4, 0.4)	1.0 (0.1, 1.9)*
	15	19.6 ± 2.5	20.6 ± 3.4	21.1 ± 3.3	20.2 ± 3.2	1.0 (0.2, 1.8)*	0.5 (−0.4, 1.4)	−0.9 (−1.8, 0.0)	0.6 (−0.3, 1.5)
	16	19.7 ± 2.9	20.4 ± 3.1	21.3 ± 3.2	20.6 ± 3.5	0.7 (−0.1, 1.5)	0.9 (0.0, 1.8)*	−0.7 (−1.6, 0.2)	0.9 (0.0, 1.8)*
	17	20.1 ± 3.0	20.6 ± 3.4	21.1 ± 3.8	20.4 ± 3.3	0.5 (−0.3, 1.3)	0.5 (−0.3, 1.3)	−0.7 (−1.5, 0.1)	0.3 (−0.5, 1.1)
	18	19.6 ± 2.4	20.4 ± 3.1	21.1 ± 3.1	20.6 ± 3.2	0.8 (−0.1, 1.6)	0.7 (−0.1, 1.5)	−0.5 (−1.4, 0.4)	1.0 (0.1, 1.9)*
	Total	18.1 ± 3.3	18.6 ± 3.6	19.0 ± 3.8	18.6 ± 3.6	0.5 (0.3, 0.8)***	0.4 (0.2, 0.7)***	−0.4 (−0.7, −0.1)***	0.5 (0.3, 0.8)***

Two-way analysis of variance with age and wave as main factors, and age-by-wave as the interaction. Bonferroni post hoc test was used for multiple comparisons. * represents *p* < 0.05, ** represents *p* < 0.01, and *** represents *p* < 0.001. M, mean; SD, standard deviation; CI, confidence interval.

## Discussion

This study used representative anthropometric data from Macao between 2005–2020 and found that (a) the average height among boys and girls increased by 2.1 cm and 2.4 cm, respectively, with downward trends in height, (b) the average weight among boys and girls increased by 4.0 kg and 2.6 kg, respectively, with downward trends for girls and stabled trends for boys, and (c) the average BMI among boys and girls increased by 1.1 kg/m^2^ and 0.5 kg/m^2^, respectively with downward or even reversed trends for girls and stabled trends for boys. The different trends in weight indicators for boys and girls over the past five years require the government, relevant departments, and schools to adopt targeted intervention strategies to improve the weight status of boys and girls and prevent nutritional problems.

During this entire period, the height, weight and BMI among Macao children and adolescents increased significantly, which was consistent with previous findings from China [9–13]. The Chinese National Survey on Students’ Constitution and Health (CNSSCH) reported that the height, weight, and BMI among Chinese children and adolescents aged 7–18 years increased by 7.0 cm, 8.0 kg, and 2.0 kg/m^2^, respectively, from 1985 to 2014 [9], and continued to increase by 2019 [11]. Similar positive trends were observed in different regions of China [10,12]. Choi et al. [16] reported combined height and weight data for children and adolescents aged 6–18 years in Macao. This study provided more detailed data, statistical estimates, and additional BMI reports. These data offer a comprehensive insight into the growth and development trends of children and adolescents in Macao. Trends in developing countries appear to differ from those in developed countries. From 1999 to 2018, there were no significant changes in height, weight, and BMI for most age groups among children and adolescents aged 6–19 years in the United States [22]. In Russia [23] and Poland [24], positive trends in growth were observed before 2010, but the trend in height has stabilized since then, while weight and BMI continue to increase. Similarly, in Japan, another Asian country, the increase in height among adolescents has stopped since the 21st century [25]. As reported by the NCD Risk Factor Collaboration, the current positive trends in growth have shifted from Western developed countries to developing countries.

In general, the occurrence of positive trends in growth was associated with improvements in living conditions, such as economic development, increased availability and diversity of food supplies, advancements in healthcare [9,12,24]. According to World Bank data, gross domestic product (GDP) per capita in Macao increased from 25.1 thousand dollars in 2005 to 81.1 thousand dollars in 2019, but it dropped a lot in 2020 because of the pandemic [26]. Macao is the only city in China where casino gambling is legal. Macao’s GDP increased from 59 billion Macau patacas in 2002–445 billion Macao patacas in 2019, while its total gambling revenue grew from 23.5 billion Macau patacas in 2002 to approximately 300 billion Macao patacas in 2019 [27]. Additionally, tourism is another important source of income for Macao. The number of tourists visiting Macao increased from approximately 7 million in 2000–39 million in 2019 (of which 24 million were from Mainland China) [28]. Changes in dietary patterns might have contributed to this trend. The Macao Education and Youth Bureau implemented the “Milk Program” (providing free milk to students) in the first grade of preschool education since the 2004/05 academic year, and has gradually extended it to higher grades each year [29]. Keung et al. [30] also reported that the proportion of Macao students aged 6–17 years who consumed dairy products daily increased from 18.9% to 28.9% between 2009 and 2015. Macao’s healthcare system offers many welfare programs. One possible important initiative is the medical subsidies program, which has been implemented since 2009. Residents are exempt from basic medical expenses when visiting health centers, and a Hospital provides free medical services to specific groups, such as children aged 10 years and below and primary and secondary school students. Since 2006, Macao has implemented free education for 15 years (from preschool to high school) [31]. Subsequently, the “2011-2020 Ten-Year Plan for the Development of Non-Higher Education” was released, marking a significant milestone in Macao’s educational development. The plan proposes to gradually enhance the education development fund and utilize the student welfare fund to provide free education subsidies. In the 2014/15 academic year, the average education subsidies per student in Macao were 3,172 Macao Patacas, which was greater than the 1,866 Macao Patacas in the 2010/11 academic year. Financial expenditures for optimizing teacher-student ratio subsidies increased from 13.0 million Macao Patacas to 52.0 million Macao Patacas [32].

This study also found that the sex difference in height among adolescents aged 18 years increased from 12.8 cm to 13.0 cm between 2005 and 2020. The CNSSCH reported that the sex difference in height among urban adolescents aged 18 years in China increased from 12.2 cm in 2000 to 12.6 cm in 2019 [12]; while in rural areas, it increased from 12.0 cm in 2005 to 12.5 cm in 2019 [11], and their increases continued. The sex difference in height observed in this study was greater than the average sex difference in adult height across 169 countries summarized by Bogin et al. in 2016 (11.8 cm) [33], but it was smaller than those in some countries that have stopped growing in recent decades (Netherlands: 13.1 cm; Denmark: 15.1 cm; Norway: 13.0 cm) [34]. The widening sex difference in height is an important manifestation of the positive long-term trend. The longer the duration of the long-term trends, the greater the sex difference in adult height. The mechanism may be that there are sex differences in growth potential determined by genetic factors. During physical growth, in conditions of poor nutrition, disease burden, and workload, the negative impact is greater for males than for females [12,35]. That is, without additional intervention, sex differences in height narrow under adverse conditions and widen under favorable conditions. Additionally, from the changes in long-term trends, the greatest increases in height occurred in the first five years for both sexes, indicating a slowing trend in height. The same trends have been observed in mainland China [10–12], possibly because they are approaching the full realization of genetic potential, and the benefits of economic growth and improved living conditions are diminishing [12,34,35]. Genetic factors contribute approximately 80% to height development [36]. On the foundation of genetically determined maximum height, environmental factors, particularly improvements in socioeconomic conditions, jointly influence an individual’s final height. Epidemiological studies indicate that favorable early nutrition and hygiene conditions help individuals reach their genetic potential, whereas adverse conditions may lead to growth restriction, preventing the realization of genetic potential [37]. Consequently, since industrialization, countries have experienced significant generational increases in average height as living standards and public health improved. However, as environmental conditions continued to improve, this growth began to slow. When overall societal nutrition and living conditions improved to the level where they no longer significantly constrained height growth, individual genetic potential became the determining factor. The population average height approached the genetic limit, entering a secular trend plateau [2,4]. In emerging economies like China, where socioeconomic conditions continue to improve, average height has continued to increase in recent years [12,34,35]. However, the growth patterns of developing countries are transitioning toward those of Western industrialized countries, and population trends in height are entering a plateau. Furthermore, the greatest increases in height occurred at the age of 12 for boys and 11 for girls, which may be related to the earlier biological maturation [10,12]. From 2005 to 2019, the median age at menarche for girls in China decreased from 12.7 years to 12.0 years, and the age at spermarche for boys decreased from 14.1 years to 13.9 years [38,39]. Unfortunately, there are no data on the maturity of Macao children and adolescents, and the data from mainland China only provide a reference.

Both boys and girls experienced significant increases in weight and BMI before 2015; however, over the past five years, their trends were opposite (continued increases in boys but decreases in girls). Chen et al. [40] reported that the prevalence of overweight and obesity among boys in Macao continued to increase from 20.2% in 2005 to 33.0% in 2020, while among girls, it increased from 14.0% to 20.9% in 2015 and then decreased to 19.6%. Tu et al. [41] reported that the prevalence of malnutrition decreased for both sexes during the same period, but from 2015 to 2020, it increased by 0.1 and 0.7 times for boys and girls, respectively, with a large rebound among girls. These trends in prevalence are almost consistent with the trends in weight and BMI observed in this study. Insufficient physical activity may lead to excessive increases in weight and BMI [9,10,12]. According to the World Health Organization’s recommendations, children aged 5–17 years should engage in at least 60 minutes of moderate-to-vigorous physical activity daily to maintain health [42]. However, researchers found that Macao adolescents engage in insufficient physical activity, with schools providing a maximum of 120 minutes of low-intensity physical activity per week; students remain sedentary for approximately 70% of their school hours [43]. Researchers speculate that this sex difference may be because Macao girls have a higher awareness of overweight, obesity, and health problems, so they pay more attention to healthy eating and exercise, and are more concerned about their health and body shape, which may reduce the increase in weight [40]. The prevalence of advertising and marketing, coupled with parents’ misunderstandings about their children’s weight, may lead girls to believe they need to lose weight even if their weight is normal; in contrast, boys rarely consider weight loss even if they are overweight [13]. A 2021 survey of secondary school students in Macao revealed that girls were more likely to overestimate their body weight. Among those who were underweight but perceived themselves as overweight, girls accounted for 26.3%, while boys accounted for only 6.8%. Among those with normal weight who perceived themselves as overweight, girls accounted for 67.7%, while boys accounted for 39.1% [44]. Additionally, the sustained gain of extra weight in boys may be related to their preference for high-energy foods such as meat and fried foods, as well as their greater intake of such foods [45].

Changes in weight and BMI among boys and girls from 2015 to 2020 also may be related to the COVID-19 pandemic. The impact of various factors varies by sex. Since December 2019, the COVID-19 pandemic has spread globally. Measurements for Macao students in 2020 were conducted between September and November, approximately 10 months after the pandemic began to have a widespread impact on society. During the COVID-19 pandemic, the Macao government implemented mandatory preventive measures, including home quarantines, lockdowns, and school closures. These restrictions harmed children and adolescents’ physical activity and dietary habits, particularly among boys, leading to weight gain [46]. Several studies have found that children and adolescents generally experienced increases in weight and BMI during COVID-19 lockdowns, with notable sex differences. Generally, boys tended to gain more weight than girls [46,47]. Compared to girls, strict lockdowns significantly eroded existing physical activity advantages among boys while exacerbating sedentary behavior disadvantages, leading to substantial declines in daily energy expenditure. Additionally, boys exhibited relatively less body image concerns and were more inclined to consume high-calorie and ultra-processed foods, resulting in increased energy intake [46,47]. Greater body image anxiety and emotional reactivity among girls may have prompted them to engage in restrictive or irregular dietary patterns [48]. Several systematic reviews reported that girls were more anxious than boys during and after the pandemic [49,50], which may have led to a decrease in food intake and subsequent weight loss. A potential explanation for sex differences in pandemic-related mental health changes may be that girls are more prone to rumination than boys, exhibit greater biological vulnerability, feel lonelier in lockdown settings, and suffer greater exposure to adversity and violence. Consequently, girls were more likely to develop internalizing symptoms (i.e., depression and anxiety) [49,50]. Therefore, in the post-pandemic era, efforts should be made to strengthen physical and mental health education, reduce and eliminate unhealthy lifestyle habits developed during the pandemic, and encourage students to engage in physical activity. Families and schools should pay attention to the diets of children and adolescents, ensuring a balanced intake of various nutrients to prevent all forms of malnutrition.

Physical measurements in the 2005, 2010, and 2015 waves of this study were concentrated between January and April, while the 2020 wave was concentrated between September and November. When interpreting differences in height, weight, and BMI across waves, it is essential to consider the potential systematic bias caused by inconsistent measurement seasons. In a Danish study of high-frequency repeated measurements among children aged 8–11 years, the growth pace of height was greater than the annual average from January to April, while fat-related indicators showed a window of accelerated growth above the annual average from late autumn to winter (approximately November to March) [51]. Monthly analysis of electronic health records from Wisconsin, USA, revealed rapid increases in children’s BMI z-scores from August to September, indicating that vacation periods represent a critical window for BMI gain [52]. Another study from the same region, which used more frequent measurements for modeling, showed that the most significant increase in BMI z-scores occurred from July to September [53]. The seasonal patterns of children’s growth and development are influenced by multiple factors, including natural environmental changes such as photoperiod, temperature, and humidity, as well as changes in individual living environments (e.g., school terms and vacations) [52–54]. Based on this, when directly comparing the 2020 wave with earlier waves in this study, bias is more likely to manifest as follows: autumn measurements occur during the annual rapid weight gain phase, leading to potentially greater weight-related indicators in the 2020 wave compared to measurements taken between January and April of the same year. Although linear height growth accelerates in spring, annual height increases remain persistent. Height levels measured in autumn may be relatively higher, potentially exaggerating the trend of the 2020 wave compared to earlier waves in wave comparisons. Nevertheless, this study revealed that anthropometric increments in a recent period (2015−2020) were nearly the smallest observed across periods. When excluding potential seasonal errors, actual increments may be even smaller, minimally affecting the interpretation of trends. However, previous research has indicated that the COVID-19 pandemic may have disrupted seasonal fluctuations in childhood growth, resulting in a greater increase in spring BMI compared to usual years [55]. Future monitoring should ideally be conducted in the same months. Alternatively, when individual-level data are available, monthly standardization and sensitivity analyses should be performed to quantify this bias. This study used representative continuous survey data from whole Macao to reveal the magnitude and pace of secular trends in height, weight and BMI among children and adolescents from 2005 to 2020. Additionally, sex- and age-stratified analyses provided comprehensive insights into the characteristics of secular trends within the study population. This study supplemented anthropometric data in the Macao region and enriched human growth research in China and developing countries.

### Limitations

This study has several limitations. First, although the majority of Macao’s population is of Han Chinese descent, other ethnic groups are also present. The trends in growth across ethnic groups may vary, and the results cannot control for ethnic factors. Second, the growth and development of adolescents may be influenced by ethnicity, biological maturity, family socioeconomic status, and intergenerational factors, which were not controlled in this study. Third, The measurement months for each wave of this study were not entirely consistent: the 2005–2015 waves were primarily measured from January to April, while the 2020 wave was concentrated from September to November. Seasonal patterns exist in children’s growth and development. Different measurement seasons may introduce systematic biases in cross-sectional comparisons and affect the attribution of differences between the 2015–2020 waves (including pandemic impacts). Since this study utilized publicly available aggregated data and could not incorporate monthly or seasonal covariates at the individual level for adjustment, findings should be interpreted with caution. Finally, the latest survey wave was conducted during the pandemic, and anthropometric data were not available for each year. Estimates of secular trends could not control for the impact of the pandemic. Continuous monitoring of anthropometry will be necessary in the future to investigate the impact of the pandemic.

## Conclusion

In conclusion, there were positive long-term trends in height, weight and BMI among Macao children and adolescents aged 6–18 years from 2005 to 2020. The trends in height for both sexes have declined. The weight and BMI of boys have continued to increase. The weight and BMI of girls continued to increase until 2015, and thereafter declined. However, the 2020 trends must be interpreted with caution due to the confounding effects of both the pandemic and the inconsistent measurement seasons. The Macao government needs to continue implementing health interventions to ensure full realization of genetic potential in height among children and adolescents. Moreover, it is necessary to focus on excessive weight gain for boys and loss for girls and actively implement post-pandemic health prevention measures, such as physical education and nutrition interventions in schools, family, and community, to cultivate healthy lifestyle habits and prevent the nutritional problems.

## Supporting information

S1 Table Sample sizes among Macao children and adolescents by test, wave, sex and age. (DOC)
