# Supplementary material for: Long-term trends in height, weight and body mass index of children and adolescents in Macao Special Administrative Region (China), 2005–2020
Source: PLoS One. 2026 Jun 12;21(6):e0351677. doi: 10.1371/journal.pone.0351677 (PMC13262830; doi:10.1371/journal.pone.0351677)
Supplement: S1 Table — (DOC) [file pone.0351677.s001.doc]

S1 Table. Sample sizes among Macao children and adolescents by test, wave, sex and age.

| Test | Age (years) | Boys | | | | Girls | | | |
| --- | --- | --- | --- | --- | --- | --- | --- | --- | --- |
|  |  | 2005 | 2010 | 2015 | 2020 | 2005 | 2010 | 2015 | 2020 |
| Height (cm) | 6 | 173 | 104 | 180 | 238 | 155 | 94 | 140 | 171 |
|  | 7 | 222 | 201 | 238 | 238 | 165 | 159 | 185 | 172 |
|  | 8 | 196 | 172 | 187 | 257 | 150 | 146 | 132 | 157 |
|  | 9 | 193 | 202 | 218 | 188 | 165 | 155 | 142 | 160 |
|  | 10 | 185 | 173 | 170 | 198 | 163 | 147 | 148 | 142 |
|  | 11 | 176 | 149 | 171 | 180 | 151 | 151 | 149 | 151 |
|  | 12 | 188 | 196 | 190 | 207 | 172 | 175 | 141 | 185 |
|  | 13 | 178 | 185 | 181 | 200 | 164 | 159 | 124 | 155 |
|  | 14 | 182 | 162 | 199 | 186 | 151 | 176 | 144 | 151 |
|  | 15 | 179 | 188 | 184 | 169 | 198 | 169 | 158 | 152 |
|  | 16 | 174 | 162 | 200 | 174 | 184 | 187 | 150 | 158 |
|  | 17 | 166 | 186 | 214 | 184 | 167 | 203 | 162 | 200 |
|  | 18 | 162 | 143 | 163 | 163 | 159 | 186 | 174 | 156 |
|  | Total | 2374 | 2223 | 2495 | 2582 | 2144 | 2107 | 1949 | 2110 |
| Weight (kg) | 6 | 173 | 104 | 180 | 238 | 155 | 94 | 140 | 171 |
|  | 7 | 222 | 201 | 238 | 238 | 165 | 159 | 185 | 172 |
|  | 8 | 196 | 172 | 188 | 257 | 150 | 146 | 132 | 157 |
|  | 9 | 193 | 202 | 218 | 188 | 165 | 155 | 142 | 160 |
|  | 10 | 185 | 173 | 170 | 199 | 163 | 147 | 148 | 142 |
|  | 11 | 176 | 149 | 170 | 180 | 151 | 151 | 149 | 151 |
|  | 12 | 188 | 196 | 190 | 208 | 172 | 175 | 141 | 185 |
|  | 13 | 178 | 185 | 181 | 200 | 164 | 159 | 124 | 155 |
|  | 14 | 182 | 162 | 199 | 186 | 151 | 176 | 143 | 151 |
|  | 15 | 179 | 188 | 183 | 169 | 198 | 168 | 158 | 152 |
|  | 16 | 174 | 162 | 200 | 174 | 184 | 187 | 150 | 158 |
|  | 17 | 166 | 186 | 214 | 184 | 167 | 202 | 162 | 200 |
|  | 18 | 162 | 143 | 163 | 163 | 159 | 186 | 174 | 156 |
|  | Total | 2374 | 2223 | 2494 | 2584 | 2144 | 2105 | 1948 | 2110 |
| Body mass index (kg/m^2^) | 6 | 173 | 104 | 180 | 238 | 155 | 94 | 140 | 171 |
|  | 7 | 222 | 201 | 238 | 238 | 165 | 159 | 185 | 172 |
|  | 8 | 196 | 172 | 187 | 257 | 150 | 146 | 132 | 157 |
|  | 9 | 193 | 202 | 218 | 188 | 165 | 155 | 142 | 160 |
|  | 10 | 185 | 173 | 170 | 198 | 163 | 147 | 148 | 142 |
|  | 11 | 176 | 149 | 170 | 180 | 151 | 151 | 149 | 151 |
|  | 12 | 188 | 196 | 190 | 207 | 172 | 175 | 141 | 185 |
|  | 13 | 178 | 185 | 181 | 200 | 164 | 159 | 124 | 155 |
|  | 14 | 182 | 162 | 199 | 186 | 151 | 176 | 143 | 151 |
|  | 15 | 179 | 188 | 183 | 169 | 198 | 168 | 158 | 152 |
|  | 16 | 174 | 162 | 200 | 174 | 184 | 187 | 150 | 158 |
|  | 17 | 166 | 186 | 214 | 184 | 167 | 202 | 162 | 200 |
|  | 18 | 162 | 143 | 163 | 163 | 159 | 186 | 174 | 156 |
|  | Total | 2374 | 2223 | 2493 | 2582 | 2144 | 2105 | 1948 | 2110 |
